# Electronic Structure and Transport Properties of Bi_2_Te_3_ and Bi_2_Se_3_ Single Crystals

**DOI:** 10.3390/mi14101888

**Published:** 2023-09-30

**Authors:** Vyacheslav V. Marchenkov, Alexey V. Lukoyanov, Semyon T. Baidak, Alexandra N. Perevalova, Bogdan M. Fominykh, Sergey V. Naumov, Elena B. Marchenkova

**Affiliations:** 1 M.N. Mikheev Institute of Metal Physics of Ural Branch of Russian Academy of Sciences, 620108 Ekaterinburg, Russia; march@imp.uran.ru (V.V.M.); baidak@imp.uran.ru (S.T.B.); domozhirova@imp.uran.ru (A.N.P.); fominykh@imp.uran.ru (B.M.F.); naumov@imp.uran.ru (S.V.N.); emarchenkova@imp.uran.ru (E.B.M.); 2 Institute of Physics and Technology, Ural Federal University Named after the First President of Russia B.N. Yeltsin, 620002 Ekaterinburg, Russia

**Keywords:** 2D materials, topological insulator, Bi_2_Te_3_, Bi_2_Se_3_, electronic structure, DFT, materials informatics, topological resistivity, Hall effect, current carrier concentration

## Abstract

The electrical resistivity and the Hall effect of topological insulator Bi_2_Te_3_ and Bi_2_Se_3_ single crystals were studied in the temperature range from 4.2 to 300 K and in magnetic fields up to 10 T. Theoretical calculations of the electronic structure of these compounds were carried out in density functional approach, taking into account spin–orbit coupling and crystal structure data for temperatures of 5, 50 and 300 K. A clear correlation was found between the density of electronic states at the Fermi level and the current carrier concentration. In the case of Bi_2_Te_3_, the density of states at the Fermi level and the current carrier concentration increase with increasing temperature, from 0.296 states eV^−1^ cell^−1^ (5 K) to 0.307 states eV^−1^ cell^−1^ (300 K) and from 0.9 × 10^19^ cm^−3^ (5 K) to 2.6 × 10^19^ cm^−3^ (300 K), respectively. On the contrary, in the case of Bi_2_Se_3_, the density of states decreases with increasing temperature, from 0.201 states eV^−1^ cell^−1^ (5 K) to 0.198 states eV^−1^ cell^−1^ (300 K), and, as a consequence, the charge carrier concentration also decreases from 2.94 × 10^19^ cm^−3^ (5 K) to 2.81 × 10^19^ cm^−3^ (300 K).

## 1. Introduction

The quantum Hall effect, in which the Hall conductivity of a two-dimensional insulator in a high magnetic field is quantized, is one of the important discoveries in condensed matter physics [1]. Special conducting edge states appear in the material in the quantum Hall effect regime. This effect is shown to have a topological nature, and such edge states can be associated with a topological invariant called the Chern number [2,3]. A nonzero Chern number determines the presence of conducting edge states, and a zero Chern number means an insulating state in the bulk, which is observed in the quantum Hall effect. Thus, topological materials can be considered as a special state of matter at the intersection of real materials and abstract mathematical topology. Such materials include topological insulators and topological semimetals. The quantum Hall effect can be considered the first two-dimensional topological insulator. Then, three-dimensional topological insulators were theoretically predicted [4,5] and experimentally discovered [6,7]. Recently, Dirac and Weyl topological semimetals were discovered [8,9,10,11,12].

A topological insulator is an insulator or semiconductor in bulk, whereas a special quantum state of electrons occurs on its surface, which makes charge carriers “topologically protected” from scattering. Such surface states are analogues of the edge states in the quantum Hall effect, and the spin–orbit coupling plays a role of the magnetic field. The metallic surface states of a topological insulator are called Dirac cones, which can be assigned a nonzero Chern number that determines the nontrivial topology of the band structure [5,8,9].

Bi_2_Te_3_ and Bi_2_Se_3_ are typical representatives of the family of topological insulators [13,14]. With the help of external influences (magnetic field, temperature, pressure, etc.), one can fine-tune their electronic structure and, consequently, purposefully change their physical properties. This, in turn, can be used in various devices. Due to their special surface states, Bi_2_Te_3_ and Bi_2_Se_3_ have great application potential and are successfully used in spintronic [15,16,17,18] and thermoelectronic [19,20,21,22,23] devices, biological and chemical sensors [24,25,26], and photonic and optoelectric applications [27,28]. Therefore, obtaining new information about the features of the electronic structure and electronic transport in such topological materials is of great interest and is relevant from both fundamental and applied points of view.

Despite the qualitatively similar electronic structure of Bi_2_Te_3_ and Bi_2_Se_3_, there are differences the band gap, the energy position of Dirac points on the surface band spectrum, and the strength of spin–orbit coupling. Taking these into account leads to a decrease/increase in the band gap in Bi_2_Te_3_/Bi_2_Se_3_, respectively; see, for example, [13,16,29,30,31]. All this inevitably manifests itself in electronic properties.

The density of electronic states at the Fermi level *N*(*E_F_*) is one of the most important characteristics, and is closely related to many electronic characteristics, particularly the current carrier concentration *n*. In [32,33], the Hall effect was experimentally studied in Bi_2_Te_3_ and Bi_2_Se_3_, and it was shown that the current carrier concentration *n* varies with temperature in different ways: it increases with temperature in the case of Bi_2_Te_3_ [32] and almost does not depend on temperature for Bi_2_Se_3_ [33]. One possible reason for this difference in the behavior of *n*(*T*) may be the different behavior of *N*(*E_F_*) with temperature. This formed the basis of this work.

The aim of this work is to establish a relationship between the density of electronic states *N*(*E_F_*) and the current carrier concentration *n* in Bi_2_Te_3_ and Bi_2_Se_3_ topological insulators. The density of electronic states and band structure were determined in the theoretical calculations using the density functional approach, considering spin–orbit coupling, and the charge carrier concentration was determined from experimental studies of the Hall effect in the temperature range from 4.2 K to 300 K in a magnetic field of 10 Т.

## 2. Materials and Methods

Topological insulator Bi_2_Te_3_ and Bi_2_Se_3_ single crystals were grown by the Bridgman–Stockbarger method. The Bi, Te, or Se components were taken in the required proportion, that is, 2:3; then these components were ground, mixed, and placed in a quartz ampoule with an elongated sharp tip. The ampoule was evacuated to a residual pressure of ~10^−4^ atm and placed in a furnace with a large temperature gradient of about 50 degrees/cm. Then, the ampoule was heated to a temperature of about 750 °C until the initial components were completely melted. The ampoule was kept for 2 h, and then it descended slowly, at a rate of ~2–5 mm/h, into the cold zone of the furnace. The single crystals grown during this process had a cylindrical shape with a sharp tip and dimensions of ~5–10 mm in diameter and ~10–20 mm in length. The crystal structure and chemical composition of the grown single crystals were studied by X-ray diffraction analysis and scanning electron microscopy at the Collaborative Access Center “Testing Center of Nanotechnology and Advanced Materials” of M.N. Mikheev Institute of Metal Physics of the Ural Branch of the Russian Academy of Sciences (IMP UB RAS).

The theoretical calculations of the electronic and band structures of Bi_2_Te_3_ and Bi_2_Se_3_ were carried out in the Quantum ESPRESSO set of computer programs [34,35]. The experimental crystal structure data were taken from the calculations for bulk unit cells of Bi_2_Te_3_ and Bi_2_Se_3_. Generalized gradient approximation within the Perdew–Burke–Ernzerhof form, usually abbreviated as PBE, for the exchange–correlation functional [36], was used for the electronic structure calculations. Spin–orbit coupling was taken into account in all calculations to provide correct band structure and band gap values, employing full relativistic ultrasoft pseudopotentials as set in the standard Quantum Espresso library of pseudopotentials [37]. A kinetic energy cutoff of 70 Ry was taken for wavefunctions, and 700 Ry for charge density and potential. A grid of 12 × 12 × 12 *k*-points was used in the first Brillouin zone for integration using the tetrahedron method. All ions were found to have no magnetic moments in the calculations.

**Figure 1:**
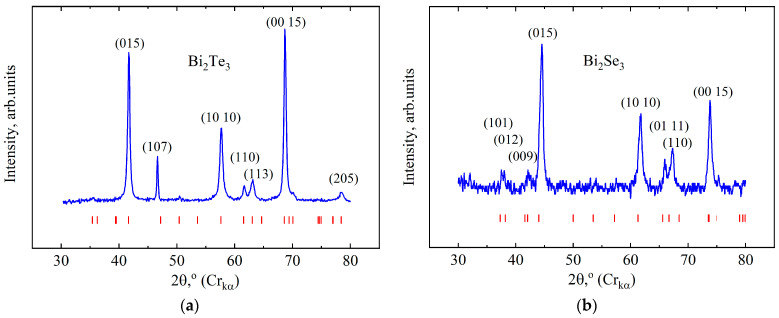
A fragment of the diffraction pattern of the Bi_2_Te_3_ (**a**) and Bi_2_Se_3_ (**b**) ground single crystals. The red dashes are the positions of the Bragg peaks.

**Figure A1:**
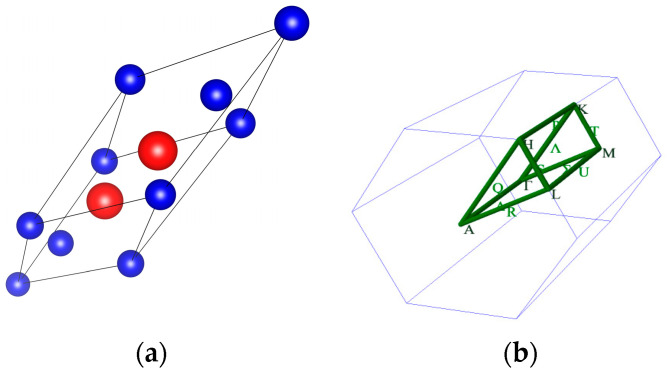
Crystal structure of Bi_2_Te_3_/Bi_2_Se_3_ (**a**) plotted in Vesta [48] with the red balls represent bismuth atoms and the blue balls represent tellurium/selenium atoms. The Brillouin zone (**b**) with high symmetry points is shown as per [49].

Figure 1 shows the X-ray diffraction patterns of the Bi_2_Te_3_ and Bi_2_Se_3_ single crystal. Bi_2_Te_3_ and Bi_2_Se_3_ single crystals were found to have a rhombohedral structure (space group *R*$\bar{3}$*m*). Figure A1a shows an image of the crystal structure of Bi_2_Te_3_ and Bi_2_Se_3_. They belong to a group of compounds that crystallize into a layered structure, the layers in which are perpendicular to the threefold symmetry axis. Using X-ray data, the lattice parameters of both single crystals were determined. The lattice parameters are *a* = 4.389 Å, *c* = 30.483 Å and *a* = 4.134 Å, *c* = 28.68 Å for Bi_2_Te_3_ and Bi_2_Se_3_, respectively (Table 1). The obtained parameters are in good agreement with the available literature data (see, for example, [38]).

**Figure A2:**
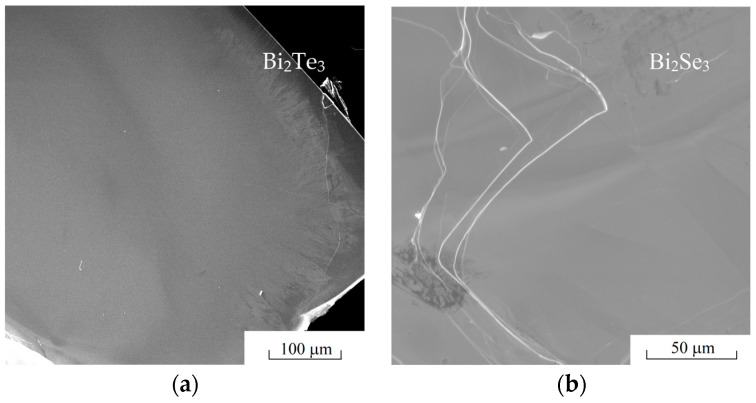
SEM images of the surface microstructure of Bi_2_Te_3_ (**a**) and Bi_2_Se_3_ (**b**).

The chemical composition of the single crystals was studied using a Tescan Mira scanning electron microscope (SEM) equipped with Oxford Instruments (Tescan Brno s.r.o., Czech Republic) INCA x-act EDS spectroscope and electron backscatter diffraction. According to the studies, the real chemical composition of the single crystals is in good agreement with the nominal one (Table 1). Figure A2 shows SEM images of the surface microstructure of Bi_2_Te_3_ and Bi_2_Se_3_, which indicate the high quality of the grown crystals and are comparable with the data presented in [39,40].

The electrical resistivity and the Hall effect were measured by the four-, and five- contact method (see, for example, [41,42]) in magnetic fields up to 10 T in the temperature range from 4.2 to 300 K using an Oxford Instruments system at the Collaborative Access Center of IMP UB RAS.

## 3. Results and Discussion

### 3.1. Band and Electronic Structures

The electronic and band structures of Bi_2_Te_3_ and Bi_2_Se_3_ were calculated theoretically in DFT-GGA approach, taking into account spin–orbit coupling which is essential to obtain the insulating band and electronic structure.

**Figure 2:**
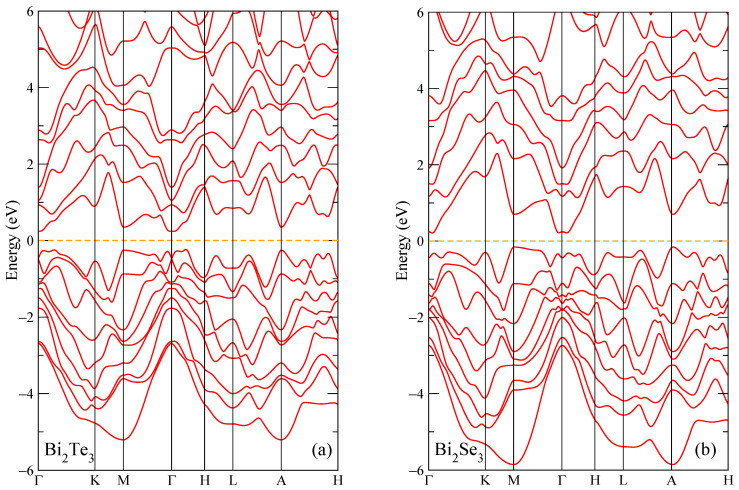
Band structure of Bi_2_Te_3_ (**a**) and Bi_2_Se_3_ (**b**). The Fermi energy is shown at zero as a horizontal dashed line.

The insulating state in both compounds is a result of the band inversion near high-symmetry point Г, which implies the presence of the surface states at the Fermi energy. One can also notice another topological feature in the band structure of Bi_2_Te_3_, Figure 2a, which is a point of band degeneration just below the Fermi level right at the high-symmetry point Г with surrounding linear dispersion. In the band structure of Bi_2_Te_3_ the bandgap was calculated as 0.48 eV; see Figure 2a. For the second compound, Bi_2_Se_3_, the bandgap in the band structure was obtained as 0.41 eV; see Figure 2b. The energy gap values and insulator state are in agreement with the previous calculations [38].

**Figure 3:**
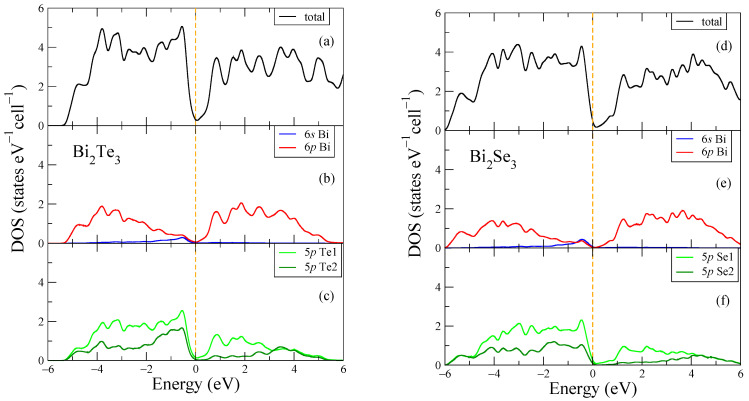
Electronic structure of Bi_2_Te_3_ (**a**–**c**) and Bi_2_Se_3_ (**d**–**f**). The Fermi energy is shown at zero as a vertical dashed line.

**Figure A3:**
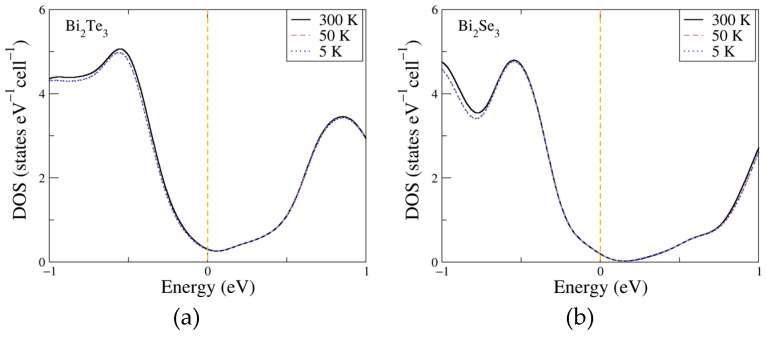
Total density of states near the Fermi level of Bi_2_Te_3_ (**a**) and Bi_2_Se_3_ (**b**) calculated for crystal structure data of 5, 50 and 300 K. The Fermi energy is shown at zero as a vertical dashed line.

From Figure 2, one can see that both Bi compounds are calculated as insulators in the band structure. However, for the plotted electronic structure shown in Figure 3, the bandgap is reproduced as a pseudogap due to the smearing procedure of the density of states (DOS) plot. The main contributions to DOS near the Fermi energy are caused by the p Bi and p Te/Se electronic states (Figure 3b,c,e,f) with the other electronic states being less represented in this energy range. For Bi_2_Se_3_, the total density of states at the Fermi energy, which is located at zero energy, was found to be equal to 0.198 states eV^−1^ cell^−1^. For Bi_2_Te_3_, the total density of states at the Fermi energy, which is located at zero energy, was found to be equal to 0.307 states eV^−1^ cell^−1^. One can notice that the bandwidth of the electronic states in Bi_2_Te_3_ is wider, and peaks are more intense than those in Bi_2_Se_3_. Similar calculations were made for the crystal structure data for low temperatures (5 and 50 K) from [38]; the results are very similar to those plotted in Figure 2 and Figure 3, for this reason are not shown, however, see Figure A3 for the DOS near the Fermi level. At the same time, the calculated values of the total density of states at the Fermi level *N*(*E_F_*) at temperatures of 5, 50 and 300 K deviate, with small differences. Below, we analyze these results in comparison with the experimental data.

### 3.2. Electronic Transport Properties

**Figure 4:**
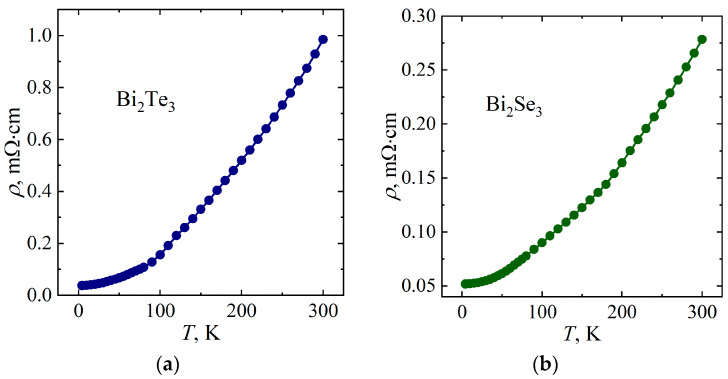
Temperature dependences of electrical resistivity of Bi_2_Te_3_ (**a**) and Bi_2_Se_3_ (**b**).

Figure 4 shows the temperature dependences of the electrical resistivity *ρ*(*T*) of Bi_2_Te_3_ and Bi_2_Se_3_ single crystals. The dependence *ρ*(*T*) is shown to have a metallic character for both samples. The residual resistivity *ρ*_0_ is 3.8 × 10^−5^ Ω·cm and 5.2 × 10^−5^ Ω·cm for Bi_2_Te_3_ and Bi_2_Se_3_, respectively. Note that the residual resistivity ratio (RRR) of the Bi_2_Te_3_ single crystal (*ρ*_300 K_/*ρ*_4.2 K_ = 26) exceeds the RRR of Bi_2_Se_3_ (*ρ*_300 K_/*ρ*_4.2 K_ = 5.4), which indicates a higher “electrical purity” of the Bi_2_Te_3_ single crystal.

**Figure 5:**
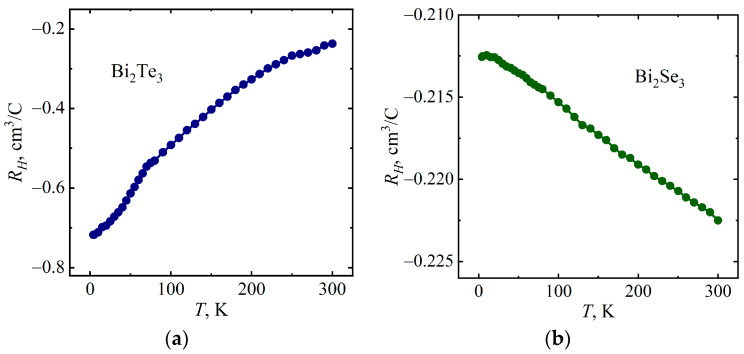
Temperature dependences of the Hall coefficient of Bi_2_Te_3_ (**a**) and Bi_2_Se_3_ (**b**).

**Figure 6:**
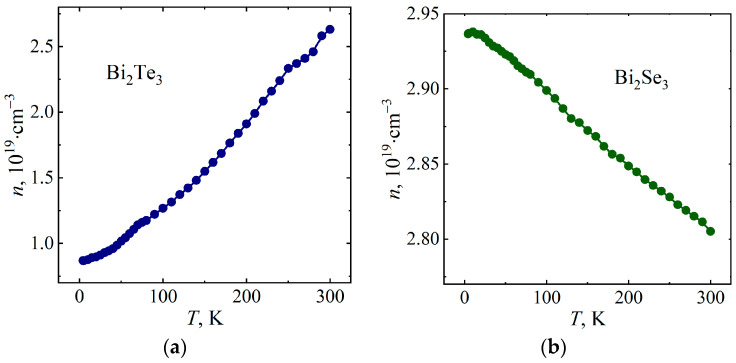
Temperature dependences of current carrier concentration in Bi_2_Te_3_ (**a**) and Bi_2_Se_3_ (**b**).

Figure 5 and Figure 6 show the temperature dependences of the Hall coefficient *R_Н_* and the current carrier concentration *n* of the Bi_2_Te_3_ and Bi_2_Se_3_ single crystals in a magnetic field *B* = 10 T, obtained from data on the Hall resistivity *ρ_xy_* in the framework of a single-band model using the following equations:

(1) $$R_{H}=\frac{\rho_{xy}}{B},$$

(2) $$n=\frac{1}{e{\cdot R}_{H}}$$

where *e* is the electron charge. Since the Hall coefficient is negative for Bi_2_Te_3_ and Bi_2_Se_3_ (Figure 5), the majority charge carriers are electrons. For Bi_2_Se_3_, one can note a slight change in the value of the current carrier concentration with temperature (Figure 6), which is consistent with previous studies [33].

**Figure 7:**
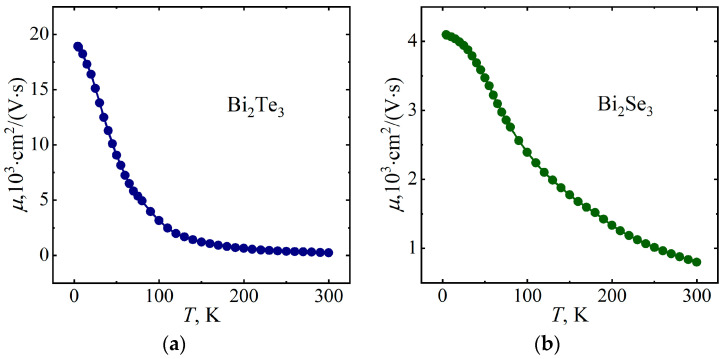
Temperature dependences of the mobility of Bi_2_Te_3_ (**a**) and Bi_2_Se_3_ (**b**).

Using the data obtained for the electrical resistivity *ρ* and the Hall coefficient *R_Н_*, the charge carrier mobilities were determined as *μ* = *R_Н_* ⁄*ρ* for Bi_2_Te_3_ and Bi_2_Se_3_ (Figure 7). The mobility is seen to decrease with increasing temperature for both Bi_2_Te_3_ and Bi_2_Se_3_, which is associated with an increase in the efficiency of current carrier scattering. The mobility is 18.9 × 10^3^ cm^2^/(V·s) and 4.1 × 10^3^ cm^2^/(V·s) at *T* = 4.2 K for Bi_2_Te_3_ and Bi_2_Se_3_, respectively. The higher value of *μ* for Bi_2_Te_3_ at low temperatures is due to the higher RRR for this single crystal compared to Bi_2_Se_3_.

A comparison of the electronic transport characteristics of bulk Bi_2_Te_3_ and Bi_2_Se_3_ single crystals obtained in this study with previously reported data for bulk crystals and thin films of Bi_2_Te_3_ and Bi_2_Se_3_ grown by other methods is given in Table 2.

### 3.3. Current Carrier Concentration Analysis

**Figure 8:**
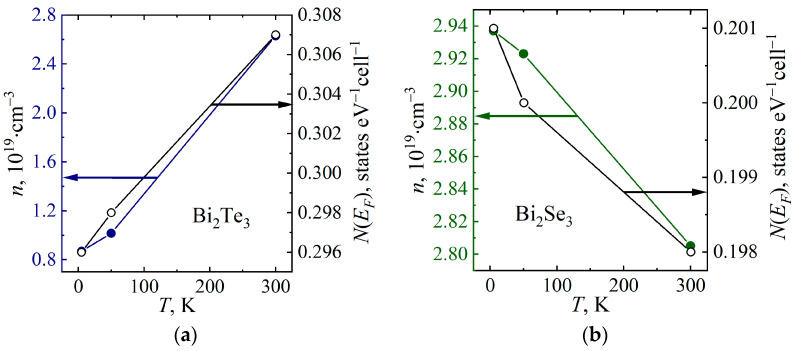
Density of states at the Fermi level *N*(*E_F_*) and current carrier concentration *n* of Bi_2_Te_3_ (**a**) and Bi_2_Se_3_ (**b**) determined at temperatures of 5 K, 50 K and 300 K. Filled circles represent the carrier concentration, open circles represent the density of states at the Fermi level.

Figure 8 shows the calculated values of the density of states at the Fermi level *N*(*E_F_*) at temperatures of 5 K, 50 K, and 300 K, as well as the charge carrier concentrations *n* determined from the experimental data at the same temperatures. As can be seen from Figure 8, there is a good correlation between the behavior of *N*(*E_F_*) and *n* with temperature for both Bi_2_Te_3_ and Bi_2_Se_3_. In the case of Bi_2_Te_3_, *N*(*E_F_*) and *n* increase with temperature, whereas in the case of Bi_2_Se_3_, *N*(*E_F_*) and *n* decrease with increasing temperature.

## 4. Conclusions

The concentrations and mobility of current carriers in topological insulator Bi_2_Te_3_ and Bi_2_Se_3_ single crystals are estimated using a single-band model -. The calculations of the band and electronic structures of Bi_2_Te_3_ and Bi_2_Se_3_ made using the density functional approach confirmed the bandgap in both compounds. It is shown that the charge carrier concentration in Bi_2_Te_3_ increases with increasing temperature, whereas the charge carrier concentration in Bi_2_Se_3_, on the contrary, slightly decreases with temperature, which is consistent with the previously reported experimental results. A good correlation has been established between the behavior of the values calculated for the density of states at the Fermi level and the charge carrier concentration determined on the basis of experimental data with temperature.

## Figures and Tables

**Table 1:** Type of crystal structure and lattice parameters of Bi_2_Te_3_ and Bi_2_Se_3_.

Compound	Crystal Structure	Lattice Parameters	Chemical Composition
Bi_2_Te_3_	Rhombohedral (space group *R*$\bar{3}$*m*)	*а* = 4.389 Å *c* = 30.483 Å	Bi_2.02_Te_2.98_
Bi_2_Se_3_	Rhombohedral (space group *R*$\bar{3}$*m*)	*а* = 4.134 Å *c* = 28.68 Å	Bi_2.01_Se_2.99_

**Table 2:** Electronic transport characteristics of Bi_2_Te_3_ and Bi_2_Se_3_.

Compound	Sample Type	Growth Method	RRR	*ρ*_0_, Ω·cm	*n*, cm^−3^ *	*μ*, cm^2^/(V·s) *	Reference
Bi_2_Te_3_	Bulk	Bridgman–Stockbarger method	26	3.8 × 10^−5^	8.70 × 10^18^	18.9 × 10^3^	This study
	Bulk	Spark plasma sintering	-	-	~2.2 × 10^19^	~10^3^	[32]
	Bulk	Self-flux method	-	~0.1 × 10^−3^	-	-	[43]
	Film	Metal organic chemical vapor deposition	~2.55	~1.35 × 10^−3^	~6 × 10^18^	~800	[44]
Bi_2_Se_3_	Bulk	Bridgman–Stockbarger method	5.4	5.2 × 10^−5^	2.94 × 10^19^	4.1 × 10^3^	This study
	Bulk	Heating stoichiometric mixtures of pure elements	-	-	~2 × 10^19^ at 100 K	~10^3^ at 100 K	[33]
	Bulk	Self-flux method	-	~0.1 × 10^−3^	-	-	[40]
	Bulk	-	~2	~0.22 × 10^−3^	~4.5 × 10^19^	~680	[45]
	Film	Vapor phase epitaxy	~2.17	0.608 × 10^−3^	~1.07 × 10^19^	954	[46]
	Nanoplate	Vapor–liquid–solid mechanism	-	-	5.2 × 10^18^	8.8 × 10^3^	[47]

* Data are given at *T* = 4.2 K (2 K), unless otherwise indicated.

## Data Availability

The data presented in this study are available on request from the corresponding author.
